# Influence of Ultrasonic Surface Rolling on Microstructure and Wear Behavior of Selective Laser Melted Ti-6Al-4V Alloy

**DOI:** 10.3390/ma10101203

**Published:** 2017-10-19

**Authors:** Zhen Wang, Zhiyu Xiao, Chuanshou Huang, Liping Wen, Weiwen Zhang

**Affiliations:** 1 National Engineering Research Center of Near-Net Shape Forming for Metallic Materials, South China University of Technology, Guangzhou 510640, China; 201510100611@mail.scut.edu.cn (Z.W.); 201620101553@mail.scut.edu.cn (C.H.); lpwen@scut.edu.cn (L.W.); mezhang@scut.edu.cn (W.Z.); 2 Guangdong Key Laboratory for Advanced Metallic Materials Processing, South China University of Technology, Guangzhou 510640, China

**Keywords:** selective laser melting, Ti-6Al-4V, ultrasonic surface rolling process, microstructure and properties, wear

## Abstract

The present article studied the effect of ultrasonic surface rolling process (USRP) on the microstructure and wear behavior of a selective laser melted Ti-6Al-4V alloy. Surface characteristics were investigated using optical microscope, nano-indentation, scanning electron microscope, transmission electron microscope and laser scanning confocal microscope. Results indicated that the thickness of pore-free surfaces increased to 100~200 μm with the increasing ultrasonic surface rolling numbers. Severe work hardening occurred in the densified layer, resulting in the formation of refined grains, dislocation walls and deformation twins. After 1000 N 6 passes, about 15.5% and 14.1% increment in surficial Nano-hardness and Vickers-hardness was obtained, respectively. The hardness decreased gradually from the top surface to the substrate. Wear tests revealed that the friction coefficient declined from 0.74 (polished surface) to 0.64 (USRP treated surface) and the wear volume reduced from 0.205 mm^−3^ to 0.195 mm^−3^. The difference in wear volume between USRP treated and polished samples increased with sliding time. The enhanced wear resistance was concluded to be associated with the improvement of hardness and shear resistance and also the inhibition of delamination initiation.

## 1. Introduction

Ti-6Al-4V is a typical α + β dual phase titanium alloy, which has a wide application in structural materials in aerospace, automotive and biomedical engineering due to its high strength-to-weight ratio, high temperature resistance, favorable corrosion resistance and biocompatibility [1,2,3]. However, it is commonly known that titanium alloy has poor friction and wear properties, often limits its application in the wear related field [4,5,6], because titanium alloy has low work hardening coefficient [7,8], low shear strength, and low protection exerted by tribo-oxides formed at the surface [9]. Therefore, in order to improve the wear resistance of titanium alloy, more and more researchers paid attention to the field of surface modification.

USRP treatment is one of simple and effective surface modification methods, which can generate intensive strain and lead to significant grain refinement in the surface with a depth of several hundred microns though a process of static extrusion and dynamic impact [10]. The modified surface can improve the wear resistance, fatigue strength and corrosion resistance effectively. Currently, the method has been used for surface modification for various metallic, such as 40 Cr steel [11], AISI 304 stainless steel [12], copper alloys [13], magnesium alloys [14] and commercially pure titanium [15]. Li et al. [16] found that frequency and load of USRP play an important role in the microstructure of HIP Ti-6Al-4V. The maximum hardened layer thickness, residual stress and best fretting wear performance were obtained under the condition of 30 kHz and 900 N; the grains were refined to a submicron grade. When the surface treated by low temperature assisted ultrasonic surface rolling, the best fretting wear properties was received at 140 °C [17]. In particular, electric pulse-assisted USRP impacted Ti-6Al-4V alloy, the ductility increased by 550%, micro-hardness increased by 64% along with the tensile strength loss of 16% [18]. The micro-hardness gradually increased to the maximum value before the pulse frequency of 250 Hz [19]. To understand the mechanical properties of material surface after USRP treatment further, the nano-indentation was adopted for determining the micro-plasticity of treated material locally due to it being a rapid, highly accurate and non-destructive testing technique [20,21,22,23]. The results indicated that the strengthening effect tends to be obvious with the distance from surface reducing.

Until now, there is no research about USRP treated selective laser melting materials. The selective laser melting (SLM) is a novel preparation method, which uses a high energy laser beam to selectively melt metal powder, directly prepares metal parts with metallurgical bonding and has excellent performance. Compared to conventional fabrication techniques, it has advantages of a reduction of production steps, high level of flexibility, high material use efficiency and near net shape production. Many investigations on SLM processing of Ti-based alloys and composites have been carried out so far [23,24], such as the influence of process parameters, strategy and heat treatment on the microstructure and mechanical properties. However, because of the characteristic of high-speed, micro-layer and micro-zone continuous melting in SLM process, the printing layer has a slight annealing effect on the forming layer; the built-material obtains high yield strength and poor tensile elongation [25,26]. The top surface always has high tensile residual stress. Besides, defects, such as splash, undercut and non-continuous melting, also exist in SLM process. They are the source of crack initiation and expansion. In order to eliminate the tensile stress and defects of SLM part, hot isostatic pressing is commonly employed to densify SLM component [27,28]. However, this method is rather complicated, costly and has a long production period, so that it is not conducive to popularization and application. As a representative of surface reinforcement methods, USRP treatment can change surface stress state from tensile to compressive residual stress, eliminate surface defects and improve mechanical properties of materials under cold working conditions [29]. More importantly, study on the densification of SLM Ti-6Al-4V alloy treated by USRP treatment has not been reported.

In this article, USRP treatment was carried out to eliminate the fabricated defects of SLM Ti-6Al-4V alloy, strengthen the surface and enhance the wear resistance. The influence of USRP parameters on microstructure was analyzed. Wear behaviors of the modification layer were investigated and the corresponding wear mechanisms were also discussed. This was intended to provide technical supports for the application of surface strengthening of SLM Ti-6Al-4V alloy.

## 2. Materials and Methods

### 2.1. Materials

**Figure 1:**
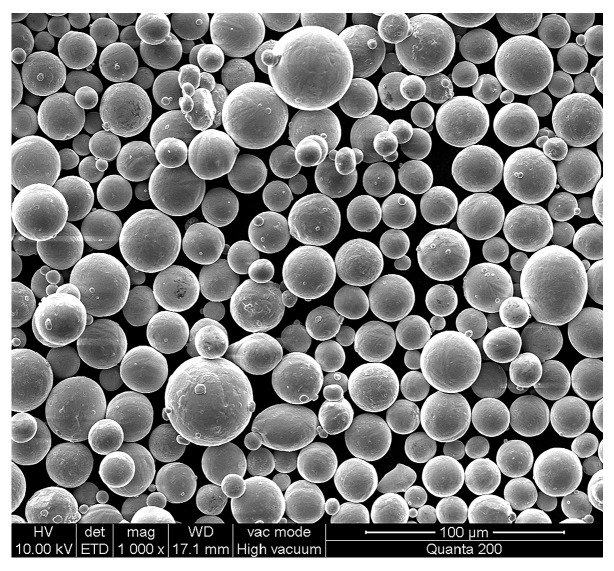
Scanning electron microscopy (SEM) image of Ti-6Al-4V alloy powders used for SLM.

Ti-6Al-4V specimens (20 mm × 20 mm × 5 mm) used in this study were prepared by selective laser melting. The alloy powder produced by Rotating Electrode Process (REP), was spherical with an average particle size of 38 μm, as shown in Figure 1. Its chemical composition was presented in Table 1. SLM was performed with following parameters: laser power was 180 W, laser scanning speed was 600 mm/s, scan line spacing was 0.07 mm and powder layer thickness was 30 μm. Density of the SLM Ti-6Al-4V alloy determined through hydrostatic weighing was approx. 4.41 g/cm^3^ that corresponds to 98% of theoretical density of cast alloy. The as-prepared Ti-6Al-4V samples were polished and then ultrasonically cleaned and dried for USRP treatment.

### 2.2. Ultrasonic Surface Rolling Process

**Figure 2:**
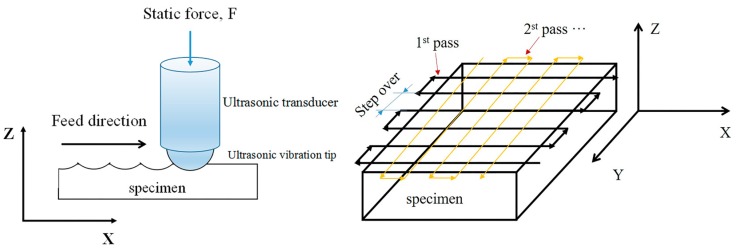
Schematic diagram of the treatment strategy.

The HK30C USRP equipment (Huayun, Jinan, China), which consisted of ultrasonic generator, piezoelectric transducer, pneumatic pump and ultrasonic vibration tip (14 mm diameter), was assembled on the vertical lathe to process the surface of the as-prepared Ti-6Al-4V alloy. Figure 2 showed the path of USRP treatment, the traverse direction of rolling ball was perpendicular to the feed direction. The basic parameters are as follows: ultrasonic vibration frequency was 30 kHz, vibration amplitude was 8 μm, normal load was 1000 N and repeated pass number was 1 and 6, respectively. Rolling line speed of vibration tip was 1 m/min, step over was 0.05 mm/step. The entire treatment was carried out at ambient temperature with coolant poured around the machining area.

### 2.3. Surface Characterization

Surface hardness was characterized by Vickers-hardness and Nano-indentation hardness. The Vickers-hardness measurements were measured using a micro-hardness instrument (MVS 1000D1, Guangjing, Guangzhou, China) with a load of 0.98 N, dwell time of 15 s; The Nano-indentation measurements were carried out using a Nano-indentation instrument (G200, Keysight, Santa Rosa, CA, USA) with diamond Berkovich indenter, which selected the dynamic nano-indentation model with the maximum indentation depth of 2500 nm. Three measured data for each specimen were averaged to reduce errors caused by different positions. To reveal the microstructure, samples were etched with a 50 mL H_2_O, 25 mL HNO_3_ and 5 mL HF solution. The cross-sectional microstructure was observed by optical microscope (DMI 5000 M, LEICA, Weitzlal, Germany) and transmission electron microscope (TECNAI G2 S-TWIN F20, FEI, Hillsboro, OR, USA). Thin TEM foils were prepared via an ion-miller (PIPS-691, Gatan, Pleasanton, CA, USA) with a beam voltage of 3.5 kV and a milling angle of 4–8°. TEM images and the corresponding selected area electron diffraction (SAED) were obtained by TEM with an accelerating voltage of 200 kV.

### 2.4. Friction and Wear Tests

The friction and wear behavior was investigated using a commercial ball-on-disk tribotester (UMT-3, CETR, Campbell, CA, USA) with 5 mm diameter silicon nitride ball under dry condition. The operating condition was kept at 5 N normal load and 8 Hz reciprocation frequency. The track of friction pair was 3 mm. The wear resistance and wear mechanism was studied by different sliding time (3, 6, 9, 15 min). Three duplicate runs were conducted for each wear condition, and then specimens were ultrasonic washed in acetone for 15 min to remove debris and impurities from worn surface. The wear volume was measured using a laser scanning confocal microscope (OLS4100, OLYMPUS, Tokyo, Japan) with 3D image acquisition function to estimate the wear resistance. The detailed operation included selecting the upper and lower thresholds and combining the separating 3D images into a complete worn morphology. The wear morphology was observed by SEM (Quanta 200 FEG, FEI, Hillsboro, OR, USA) to analyze the wear mechanism.

## 3. Result and Discussion

### 3.1. Microstructure Observation

**Figure 3:**
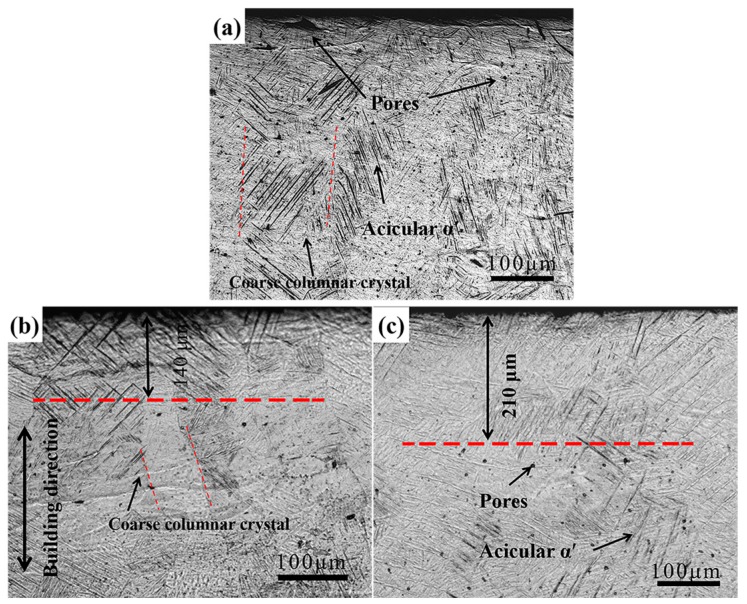
Optical images of longitudinal cross-section of (**a**) polished, (**b**) 1000 N 1 pass and (**c**) 1000 N 6 passes USRP processed SLM specimens.

Figure 3 shows the longitudinal cross-section microstructures of SLM specimens processed by USRP treatment under different conditions. Coarse columnar grains grown along the building direction could be observed due to high temperature gradients in the SLM process (Figure 3a). The average length of the columnar grain was several millimeters and the width was about 100 μm. Columnar grains consisted of fine acicular α’ martensites which were transformed from β phase as a rapid cooling effect of the laser pool [25,30]. Many small spherical pores could be seen in specimens because of the balling phenomenon, which was caused by surface tension due to the molten material, did not wet the underlying substrate [31]. In addition, there were some irregular pores with a length of about 50 μm within the surface layer, and the size was larger than spherical pores. After USRP treatment, acicular α’ matensites still existed and no phase transition happened. Meanwhile, pores were healed and a densified surface was attained. The thickness of the densified layer was about 140 μm after 1000 N 1 pass (Figure 3b), and increased to about 210 μm after 1000 N 6 passes (Figure 3c). So, the depth of the densified layer increased significantly along with pass numbers of USRP treatment.

**Figure 4:**
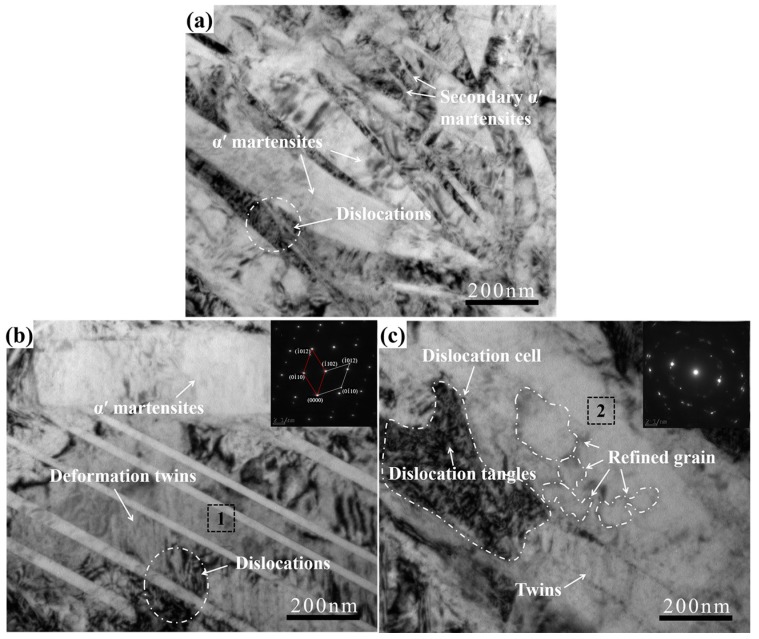
TEM images of top surface before (**a**) and after 1000 N 6 passes of USRP (**b**,**c**).

TEM examination of the microstructural evolution in the top surface of polished and USRP treated samples are shown in Figure 4. It indicated that the SLM Ti-6Al-4V alloy mainly consisted of acicular α’ martensites as seen in Figure 4a; such structures were also identified in the OM images (Figure 3). The acicular α’ martensites had a high aspect ratio and the width reached about 100~200 nm. Except for a number of acicular α’ martensites, there are numerous, fine secondary α’ martensites and chaotic arrangement of dislocations in SLM Ti-6Al-4V alloy, which was also investigated by Yang et al. [32]. After USRP treatment, these needle-like martensites could still be observed in the densified layer. Meanwhile, some deformation twins were also identified. The reason for formation of these twins is that α titanium alloy with hexagonal close packed structure had less independent slip systems and more twin planes. During the deformation process, deformation twins were created in the densified layer when the slip was block. The SAED pattern (Figure 4b) also demonstrated that the symmetrical structures along the axis were deformation twins. These deformation twins would block the movement of the dislocations, making the dislocations entangled in the twin boundaries, and resulting in a greater work hardening in the treated surface [33]. Moreover, some equiaxed sub-grains were formed near the dislocation cells as depicts in Figure 4c. The average size was evaluated at about 100~200 nm. The ring of corresponding SAED pattern reveals that these sub-grains had random crystallographic orientations. Generally, dislocation walls and dislocation cells were formed after the entanglement, aggregation, annihilation and recombination of dislocations. Some of the dislocation cells would translate into sub-grains under the action of greater strain energy consequently, which was a commonly result caused by severe work hardening [29,33].

### 3.2. Nano-Indentation and Hardness

**Figure 5:**
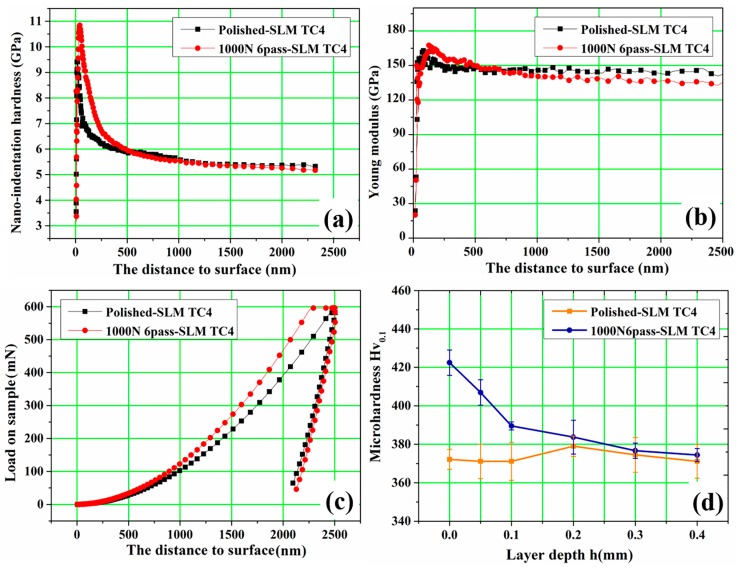
Nano-indentation test curves of the as-prepared and 1000 N 6 passes USRP treated samples on the top surface: (**a**) Nano-indentation hardness variation; (**b**) Young modulus variation; (**c**) Load variation and (**d**) Vickers hardness.

Figure 5 displays the nano-indentation test curves with respect to depth from the top surface for polished and USRP treated specimens. It could be found that the nano-indentation hardness of polished surface reached 9.5 GPa on the top surface (Figure 5a), was greater than twice that of the commercial annealed Ti-6Al-4V alloy [19], because of the harder α’-martensite phase and dislocation tangles between martensites in SLM alloy (Figure 4a). The average nano-indentation hardness increased 15.5% to 11 GPa and the Vickers-hardness increased from 370 HV to 422 HV after 1000 N 6 passes of USRP treatment. With the increase of the depth from surface, the hardness decreased gradually, and the depth of the work hardening layer was about 200 μm (Figure 5d). The increment of surface hardness of USRP treated samples was the result of work hardening, which was commonly attributed to the formation of a large number of dislocations, deformation twins, as well as the dislocation tangles occurred at the deformation twin boundary in the densified layer after USRP treatment (Figure 4b). Besides, the increment in hardness may be also ascribed to the grain refinement following the Hall-Petch relationship [33,34]. Meanwhile, USRP treatment also made the elastic modulus of a slight increase, the trend of change was similar to the nano-indentation hardness case (Figure 5b). In the load-penetration curves, the polished and USRP treated sample all needed a maximum load of 600 mn to reach a penetration depth of 2500 nm. The applied load slope of the USRP treated sample was larger than that of the polished one. These results illustrated that an excellent deformation resistance was obtained when the surface was subjected to USRP treatment.

### 3.3. Friction and Wear Behavior

#### 3.3.1. Friction Coefficient and Wear Volume

**Figure 6:**
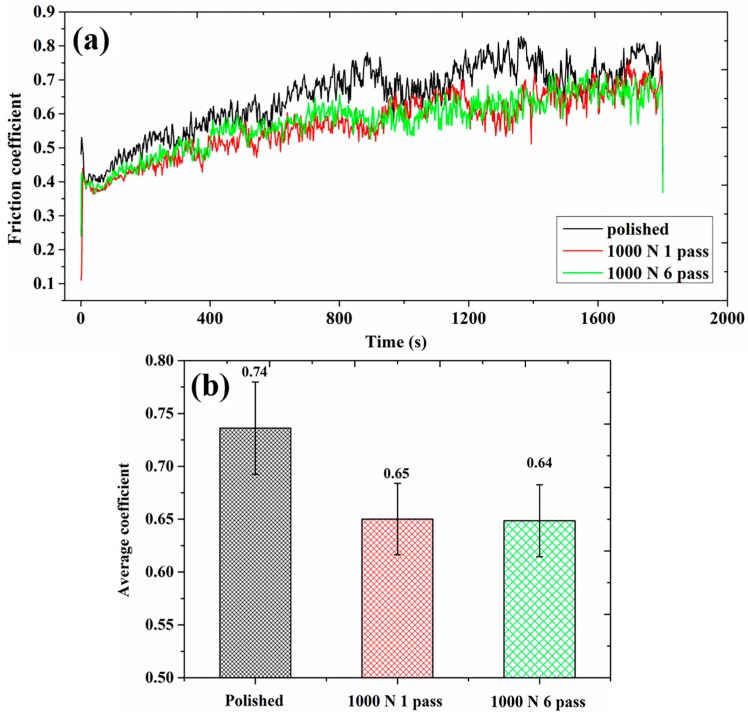
Variation of the friction coefficient of polished and USRP treated samples: (**a**) friction coefficient curves; (**b**) average coefficient.

Figure 6 shows the evolution of friction coefficient versus sliding time of USRP treated samples under different dry conditions against Si_3_N_4_ ball. It could be seen that friction coefficient curves of USRP treated samples possessed similar variation trends to that of polished sample, they all experienced a rapid running-in period (Figure 6a). Then, the friction coefficient continued to rise, and achieved a stable wear stage after about 1200 s of sliding time. Compared with the polished sample, friction coefficient of the USRP treated samples was significantly reduced with less fluctuation, the average value decreased from 0.74 to 0.64, as shown in Figure 6b. The results indicated that friction behavior could be apparently improved by USRP treatment. However, different pass numbers of USRP treatment had little effect on the friction coefficient; the average value of friction coefficient remained at 0.65 approximately.

**Figure 7:**
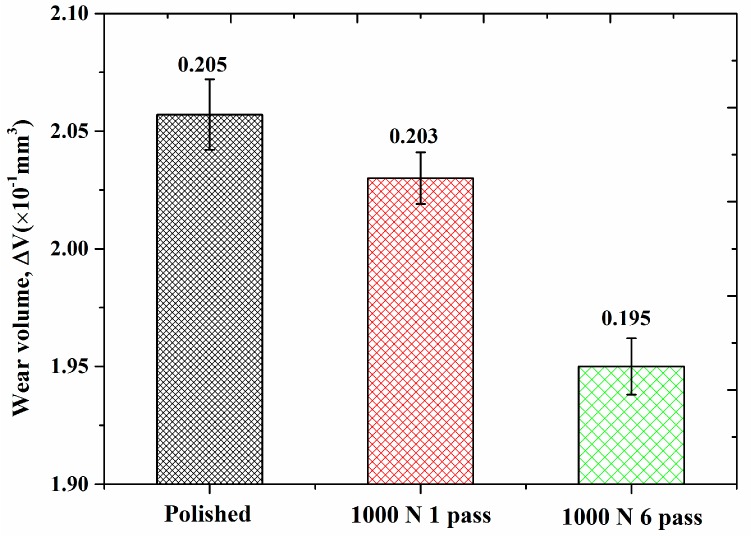
Wear volumes of SLM Ti-6Al-4V samples vs. different pass numbers.

Figure 7 presents the comparison of wear volume for polished sample and USRP treated samples under different pass numbers. The polished surface had the worst wear resistance, the wear volume was 0.205 mm^−3^; the wear resistance of samples treated by 1000 N 1 pass of USRP treatment was improved slightly, the wear volume was 0.203 m^−3^; the best wear resistance was obtained by 1000 N 6 passes of USRP treatment, the wear volume was declined to 0.195 mm^−3^. This phenomenon revealed that with the increase of pass numbers, wear volume was significantly reduced, and wear resistance was gradually improved. The highest wear volume (lowest wear resistance) was observed in the polished sample while the lowest weight loss (highest wear resistance) was obtained in the USRP treated one (1000 N 6 passes). It was well known that hardness, strength, plastic toughness and defects of material were important factors in measuring wear resistance of material. The higher the hardness, the shallower the depth of pressed material in the process of friction and wear, the less the wear volume [35]. The smaller the amount of porosity, the less the position of stress concentration source, the reduced probability of crack propagation. According to the results of Figure 5a,c,d, apparently work hardening occurred in the surface treated by USRP treatment, the hardness of the top surface was enhanced about 13~15%, and the deformation resistance of hardened surface was also improved to a certain degree. Meanwhile, the number of pores was significantly reduced, a densified layer was formed about 100~200 μm, therefore the wear resistance was effectively improved after USRP treatment.

#### 3.3.2. Analysis of Wear Mechanism

**Figure 8:**
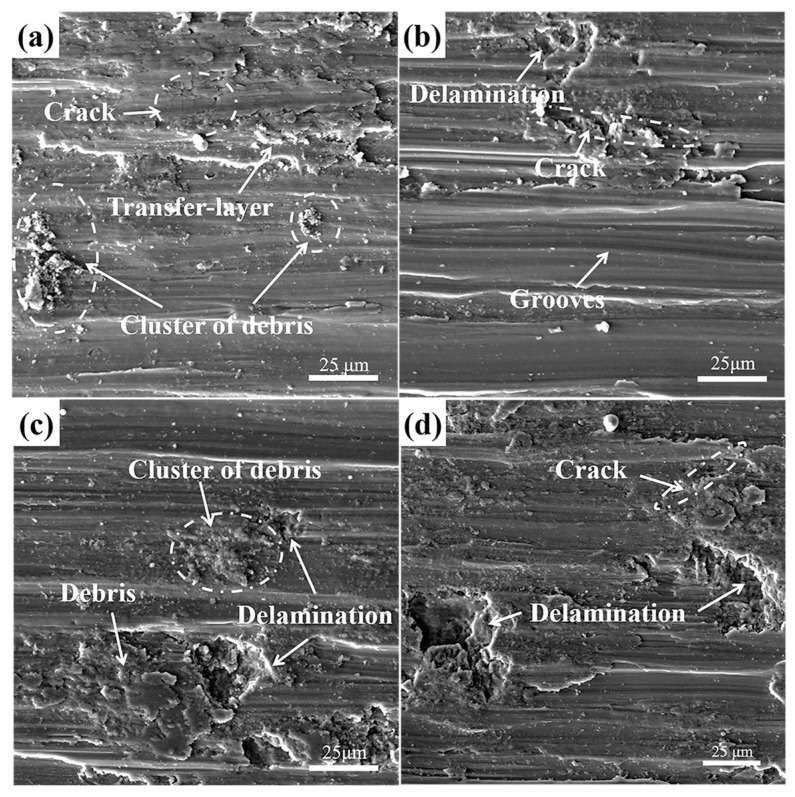
SEM images showing typical tribological features of polished Ti-6Al-4V alloy after different sliding time: (**a**) 3; (**b**) 6; (**c**) 9 and (**d**) 15 min.

Wear performance was commonly related to the microstructure and hardness of wear materials. It has been established that the microstructure of the hardening layer was different from the pristine and the hardness was a gradient distribution perpendicular to the surface. Therefore, wear performance with increasing thickness in the layer may be different. Figure 8 shows the wear morphologies of polished samples against Si_3_N_4_ ceramic ball after different sliding time. Since the specimens did not experience any specific surface treatment, the shear strength and hardness of polished samples were lower than that of USRP treated samples. The material was easily plowed from surface to form debris in the initial 3 min of sliding time. Then some debris were welded on the worn surface in the case of repeated grinding by friction pair, elongated along the sliding direction under the action of normal load. Finally, they were converted into transfer-layer as shown in Figure 8a. Meanwhile, cracks were emerged on the transfer-layer under the influence of normal load, which were perpendicular to the sliding direction. Some clusters of debris were dispersed on the worn surfaces randomly. During this period, the dominated wear mechanism was severe adhesive wear and abrasive wear. Analogous wear behavior was studied in EBM Ti-6Al-4V parts by Toh et al. [36]. When wear time reached at 6~15 min, these small debris were welded on worn surface too. Crack propagation with delamination formation could be observed on the transfer-layer. Many fine scratches also appeared in the grooves so that the interior surface was quite rough. With the sliding time increasing, delamination became more and more serious. At this stage, the dominated wear mechanism was deteriorative delamination and abrasive wear accompanied by adhesive wear.

**Figure 9:**
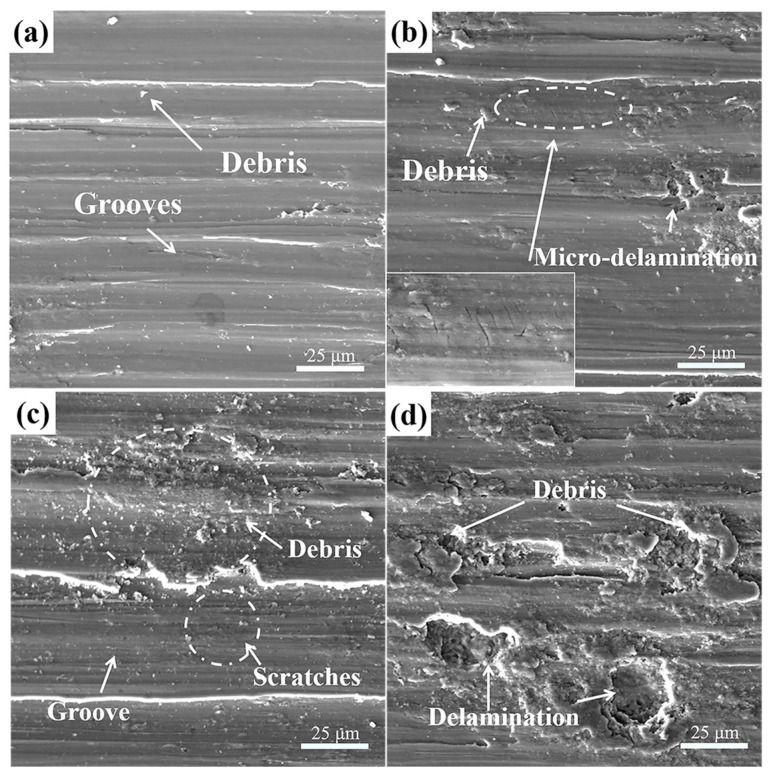
SEM images showing typical tribological features after different sliding time, observed on the worn surfaces of USRP treated Ti-6Al-4V alloy: (**a**) 3; (**b**) 6; (**c**) 9 and (**d**) 15 min.

The wear tracks on USRP treated Ti-6Al-4V alloy slide under various wear time are shown in Figure 9. When the sample was subjected to a sliding time of 3 min, worn surface showed plastic deformation as well as grooves along sliding direction. Compared with polished samples, the width of grooves was narrow and the interior surface was smooth under the circumstance. Little debris, no delamination and no micro-crack were observed around the wear tracks. This was a typical abrasive wear mechanism resulted by surface work hardening.

As the sliding time reached 6 min, the influence of work-hardening and resistance of plastic shearing declined due to gradient distribution of hardness from the surface to the sub-surface. As a consequence, the direction of cracks initiation and propagation was perpendicular to the wear tracks. Some delamination began to appear near the cracks. Meanwhile, the width of grooves was broadened obviously, the interior surface still seemed smooth and only a small amount of debris existed on the surface. When increasing the sliding time to 9 min, the quantity of debris significantly increased and some debris agglomerated to form clusters of debris. The width of the grooves were further widened. Some small scratches appeared on the interior surface, due to three-body wear caused by the presence of small-size debris. During this period, the dominant wear mechanism gradually changed from abrasive to adhesive wear.

**Figure 10:**
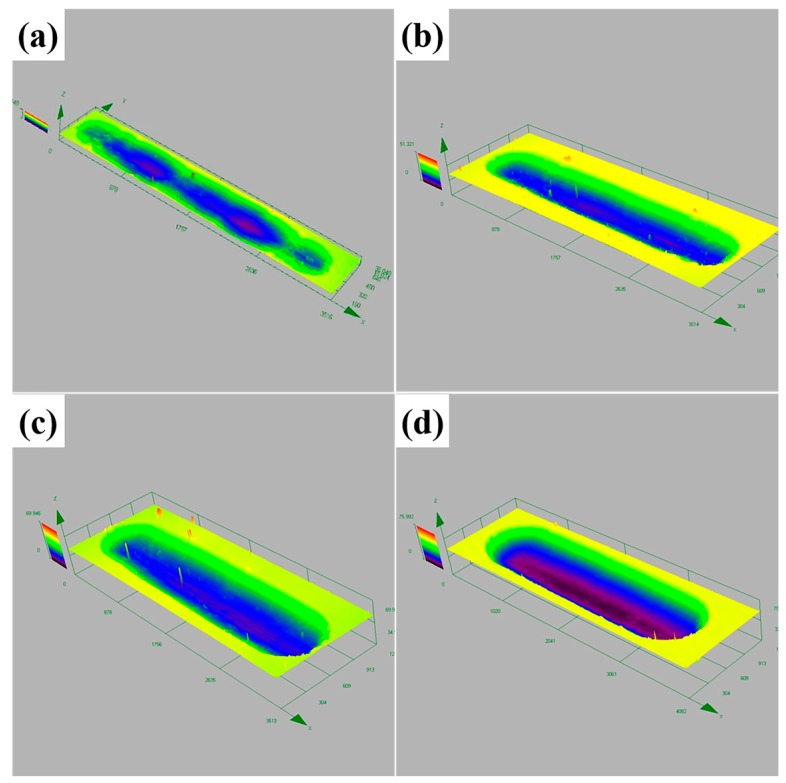
3D-profile of the wear tracks for USRP treated samples (1000 N 6 passes) under different sliding time: (**a**) 3; (**b**) 6; (**c**) 9 and (**d**) 15 min.

When sliding time reached 15 min, the volume of delamination was increased more obviously. Metal debris was detached from the worn surface more fully. The interior surface of the grooves were rougher than the former. Moreover, 3D-profile of the wear tracks for USRP treated samples revealed that the depth and width of wear tracks gradually increased with the sliding time, as shown in Figure 10. At this moment, the main wear mechanism was completely turned into delamination and serious adhesive wear.

**Figure 11:**
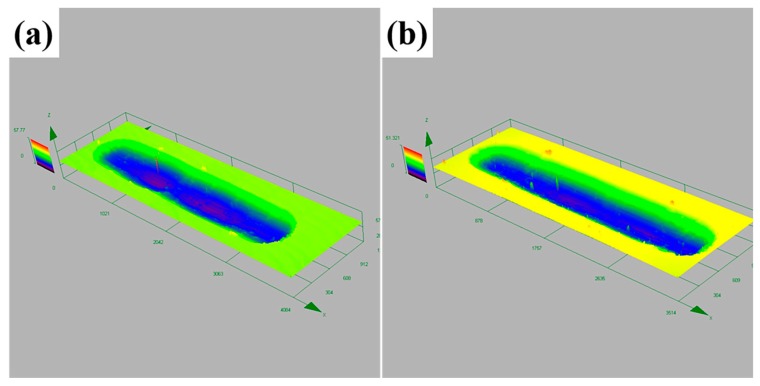
3D-profile of the wear tracks for (**a**) polished and (**b**) USRP treated titanium after 6 min sliding time.

**Figure 12:**
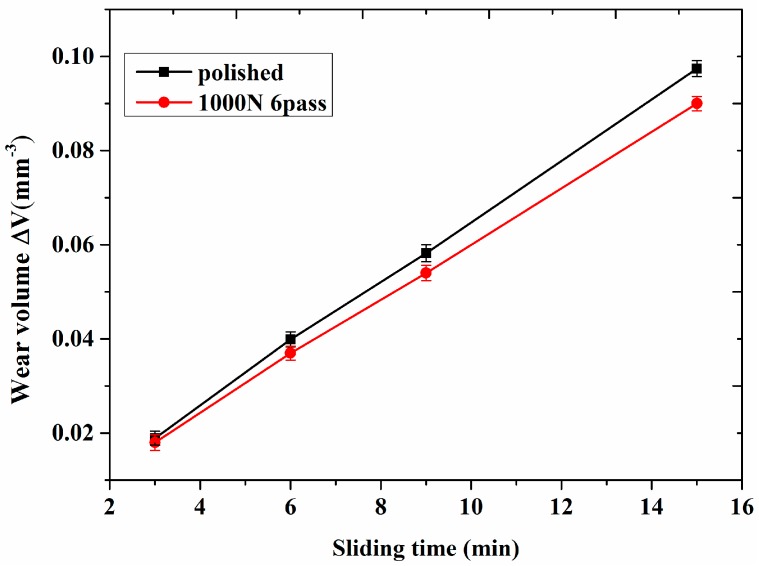
Variation of wear volume with sliding time for polished and USRP treated samples.

Figure 11 displays the 3D-profile of wear tracks for polished and USRP treated titanium after sliding time of 6 min. The various wear volume for polished and USRP treated samples changed with sliding time is shown in Figure 12. Comparing the tribological features of the worn surface before and after USRP treatment, it was visible that the worn surface of polished samples exhibited a larger amount of transferred material than those of USRP treated samples. Besides, the dominant wear mechanism of USRP treated and the polished surface was obviously different after 3~9 min of sliding time. The depth of wear track of the USRP treated sample was shallower than the polished one (Figure 11). The difference in wear volume between USRP treated samples and polished samples increased with sliding time (Figure 12). This could be attributed to the following two aspects: (1) work hardening resulted in the improvement of hardness and resistance to shear deformation (2) different degrees of delamination caused by fatigue.

Through analyzing the SEM images carefully (Figure 8 and Figure 9), it could be found that there was a time lag in tribological features between the polished and USRP treated sample. Such as, fatigue delamination was detected after 9 min for polished samples, while the delamination occurred at 15 min for USRP treated samples. So, the degree of deterioration of fatigue delamination should be one of the most important factors, which resulted in the increasing difference in wear volume between polished and USRP treated samples.

It was well known that delamination was caused by micro-crack initiation and propagation. When micro-crack spread to a critical size, shear delamination occurred parallel to the wear surface, with detached debris from surface [37]. Because slip band intersections had a high strain energy, micro-cracks often initiated in the site of slip band intersections for titanium alloy, and then propagated along {110} slip bands [38]. However, the high strain energy of slip band intersections would be largely dissipated by dislocation annihilation. In the polished surface, dislocations movement was effectively hindered by chaotic arrangement of dislocations under normal loads (Figure 4a), and these mobile dislocations could aggregate in slip planes provoking strain localization, resulting in cracks initiation in the site of slip band intersections. However, in the densified surface, those tangled dislocations annihilated and recombined to form dislocation walls, and some dislocation walls turned into small sized sub-grains under USRP treatment (Figure 4c). The dislocation annihilation and sub-grains transformation process notably declined the high strain energy of slip band intersections and prevented dislocation accumulation in the slip plans. Therefore, the dislocation annihilation and sub-grain transformation process avoided the strain localization and inhabited the micro-cracks initiation. Besides, the small sized sub-grains also decelerated the crack propagation rate as the sub-boundary formation increased the crack branching and deflection.

On the other hand, pores or pore clusters commonly acted as stress concentration sites for fatigue crack initiation [39]. Under cyclic loading, these crack initiations coalesced to form larger cracks leading to fatigue fracture. More importantly, there was a region of plastic deformation adjacent to the worn surface [40]. Pores in this region were susceptible to plastic deformation which would bring about subsurface delamination initiation at normal loads. Fortunately, these pores were healed completely in the densified surface after USRP treatment, causing the surface to possess insensitivity to the stress concentration. As a result, surface densification greatly dropped the probability of cracks nucleation in the sub-surface and controlled the delamination formation. In summary, USRP treatment could effectively improve the wear resistance of SLM titanium alloy at a low normal load, which had an important guiding role in improving the wear resistance.

## 4. Conclusions

(1) In this paper, samples of selective laser melted Ti-6Al-4V alloy were subjected to severe surface plastic deformation by using ultrasonic surface rolling process. The method could effectively increase the thickness of pore-free surface. Some substructures were formed after USRP treatment such as refined grains, dislocation wall and deformation twins, which improved the hardness and resistance to shear deformation of the modified surface.
(2) Compared friction properties and wear volumes before and after USRP treated samples. The coefficient of friction was reduced from 0.74 to 0.64, the wear volume was decreased from 0.206 mm^−3^ to 0.195 mm^−3^. As the sliding time increased, the difference of the wear volume before and after USRP treatment was gradually increased. The wear mechanism of surface was obviously changed from severe adhesive wear to abrasive wear. In the subsurface, the wear mechanism was same, which consisted of abrasion wear, adhesive and delamination.
(3) The reasons for improvement of friction and wear resistance were attributed to the increase in hardness and shear resistance as well as inhibition of delamination initiation.

## Figures and Tables

**Table 1:** Chemical composition of Ti-6Al-4V alloy powders (wt %).

Ti	Al	V	O	C
Balance	5.5~6.5	3.5~4.5	<0.13	<0.08
